# Effects of Lifestyle Interventions to Promote Physical Activity on Physical Activity and Glycated Hemoglobin in Patients with Type 2 Diabetes: a Systematic Review and Meta-Analysis

**DOI:** 10.1007/s40279-025-02184-8

**Published:** 2025-03-13

**Authors:** Vivien Hohberg, Eric Lichtenstein, Jan-Niklas Kreppke, Cedrine Zanitti, Fiona Streckmann, Markus Gerber, Oliver Faude

**Affiliations:** 1 Department of Sport, Exercise and Health, University of Basel, Gross Allee 6, 4052 Basel, Switzerland; 2 Institute of Movement and Neurosciences, German Sport University Cologne, Am Sportpark Müngersdorf 6, 50933 Cologne, Germany

## Abstract

**Background:**

Numerous studies have explored the impact of controlled exercise interventions in type 2 diabetes, as physical activity can positively influence its progression. However, our understanding of how broader lifestyle interventions can effectively promote physical activity in practical real-world scenarios remains limited.

**Objective:**

This systematic review and meta-analysis aimed to investigate the potential of lifestyle interventions targeting the promotion of physical activity on physical activity outcomes and glycated hemoglobin (HbA1c), providing a comprehensive understanding of both behavioral and clinical impacts.

**Methods:**

We performed a systematic review and meta-analysis, searching three databases and examined the study design, structure, and content of the lifestyle interventions. We assessed physical activity and HbA1c as endpoints and performed a multivariate meta-regression to explore physical activity’s impact on HbA1c.

**Results:**

This review incorporated 13 studies (*n* = 5301 patients), with heterogeneity in intervention designs, components, and durations. Lifestyle interventions showed a slight increase in physical activity, equivalent to an average of 9.0 min more total physical activity per day (95% confidence interval 5.8, 12.2) and 1.7 min more moderate-to-vigorous physical activity per day (95% confidence interval 1.1, 2.3), irrespective of objective (e.g., accelerometers) or subjective measurement (e.g., questionnaires) method. However, HbA1c reduction through these interventions was minimal 0.09% (95% confidence interval − 0.20, 0.03). The effect of physical activity was − 0.04 (standard error = 0.05, 95% confidence interval − 0.15, 0.06), suggesting that physical activity does not act as a moderator for changes in HbA1c.

**Conclusions:**

Lifestyle interventions effectively increase physical activity but have limited impact on HbA1c compared to controls. The role of physical activity as a moderator for changes in HbA1c remains uncertain. Further research is needed to enhance the efficacy of these interventions in reducing HbA1c in individuals with type 2 diabetes.

**Supplementary Information:**

The online version contains supplementary material available at 10.1007/s40279-025-02184-8.

## Key Points

**Table:** 

Lifestyle interventions in patients with type 2 diabetes lead to a slight improvement in physical activity levels.
The reduction in glycated hemoglobin levels from these interventions is minimal, indicating limited effectiveness.
There is a need for more research to understand how physical activity influences glycated hemoglobin levels, to enhance lifestyle intervention strategies.

## Introduction

Unhealthy lifestyles have contributed to an increase of type 2 diabetes (T2D) cases worldwide, affecting more than 536.6 million people in 2021 [1]. Several risk factors are associated with T2D, including physical inactivity, obesity, and weight gain [2, 3]. The prevalence of T2D is predicted to continue increasing globally, with projections indicating a rise from 10.5% in 2021 to 12.2% among adults in 2045 [1]. In addition to medical treatments, the management of T2D relies on adopting a healthy diet and regular physical activity, to better regulate glycemic control, induce weight loss, and improve maintenance. In this context, physical activity is associated with a 40–50% lower mortality risk from cardiovascular diseases (relative risk 0.55, 95% confidence interval [CI] 0.34, 0.68) and total diseases (relative risk 0.57, 95% CI 0.49, 0.67) in individuals diagnosed with T2D [4]. Although moderate-to-vigorous physical activity for 150 min per week alongside dietary modifications is typically recommended, physical activity can function as an independent treatment to prevent, delay, or even reverse T2D [5]. Incorporating habitual physical activity, at best including aerobic exercise, resistance training or a combination of these exercise modes, results in improved glycemic control [6, 7]. Structured exercise interventions have the potential to lower glycated hemoglobin (HbA1c), a marker for assessing the progression of T2D, with a weighted mean difference of − 0.28% (95% CI − 0.46, − 0.11) for moderate-intensity exercise training [8].

Lifestyle interventions should be distinguished from exercise interventions. While exercise interventions focus specifically on structured exercise programs, lifestyle interventions encompass a more comprehensive approach, targeting multiple behavioral factors beyond exercise alone. Exercise interventions are defined as structured exercise programs, which involve prescribing and implementing specific exercise regimens with the aim of improving physical fitness, muscular strength, overall physical performance, and health [9, 10]. This type of intervention has a defined duration and intensity level. A disadvantage of structured exercise interventions is that they often fail to account for the transition to real-world settings for individuals with T2D and do not consider individual preferences [11]. Furthermore, adherence to exercise programs tends to be low [12]. In contrast, lifestyle interventions extend beyond simple recommendations and education by addressing the motivational, behavioral, and emotional challenges that individuals face when making long-term lifestyle changes. These interventions provide comprehensive support to help individuals achieve and maintain their health goals [13]. The purpose of lifestyle interventions is to promote health by encouraging individuals to make health-behavioral changes in their everyday routines [14]. Lifestyle interventions are designed to target specific behavioral patterns, such as improved diet or increased physical activity, by understanding the underlying mechanisms and contexts that maintain these behaviors [15]. They are designed to meet the needs of participants and be capable of real-world impact by analyzing behavioral patterns in context. This involves applying psychological theory and evidence-based change techniques [15, 16]. This definition of lifestyle intervention aligns with the American Diabetes Association’s recommendation of person-centered care implementations [17].

Numerous studies have investigated the impact of controlled exercise interventions on T2D, as physical activity can positively influence the disease’s progression. These structured exercise interventions have shown a reduction in HbA1c, with decreases of up to − 0.71% [18–21]. However, our understanding of how lifestyle interventions can effectively promote physical activity and improve clinical outcomes like HbA1c in practical real-world scenarios remains limited. This systematic review and meta-analysis aimed to address this gap by including studies that report physical activity and HbA1c outcomes, thereby providing a comprehensive assessment of lifestyle interventions on both behavioral and clinical outcomes in T2D management. Therefore, this systematic review and meta-analysis aimed to address the following questions:

1. How do lifestyle interventions, aimed at promoting physical activity, impact on physical activity and HbA1c?
2. Is the effect of lifestyle interventions on HbA1c moderated by participants’ physical activity?

## Methods

The systematic review and meta-analysis followed the guidelines of the Preferred Reporting Items for Systematic Reviews and Meta-Analyses (PRISMA) [22]. It was a priori registered in PROSPERO (CRD42023406422). A protocol was not prepared.

### Search Strategy

Searches for appropriate studies were conducted in three databases: MEDLINE (via PubMed), PsycINFO, and SPORTdiscus (via EBSCO). The search period for each database was from January 2000 to 27 March, 2023. The search terms included a combination of “T2D” AND “lifestyle intervention” AND “randomized controlled trial” AND “physical activity” AND “hemoglobin A1C”. The full search strategy can be found in the Electronic Supplementary Material (ESM). Additionally, a manual search of the reference lists of included studies was conducted to identify all relevant studies. No gray literature or study registries were searched.

### Eligibility Criteria

The inclusion criteria for eligible studies were based on the PICOS scheme (population, intervention, comparator, outcome, study design). Studies were included if (1) the study population had T2D and was over 18 years old, (2) the study examined the effect of a lifestyle intervention as defined in the Sect. 1 (addressing motivational, behavioral, or emotional challenges, comprehensive support to help individuals achieve and maintain their health goals, targeting specific behavioral patterns, adaptability, and flexibility in terms of the real-world setting of the participants) to promote physical activity, (3) the intervention was compared to a control group (waiting list, treatment as usual, minimal intervention), (4) the studies measured self-reported or objectively measured physical activity and HbA1c as outcomes after the end of the intervention, (5) the interventions were tested in randomized controlled trials (RCTs) published in English or German since the year 2000. Studies were excluded if the study participants had psychiatric disorders, type 1 diabetes, or gestational diabetes. Furthermore, studies that did not utilize validated measurement tools to assess physical activity (e.g., non-validated questionnaires) were excluded.

### Study Selection

Duplicates of the identified studies were individually reviewed and removed using Covidence. VH and CZ independently screened the studies using the title and abstract as well as full-text screening following the predefined inclusion and exclusion criteria. Any discrepancies were discussed, and a final decision on eligibility was made through consensus among VH, CZ, and OF. In cases where the full texts of the studies were not available for screening, the corresponding authors were contacted to request access to the full text.

### Data Extraction and Outcome Parameters

Relevant details from each study were extracted by two authors. All extracted data were recorded and subsequently organized in an Excel spreadsheet for analysis (ESM).

Data on physical activity and HbA1c were extracted from each included study. Glycated hemoglobin is characterized by the percentage of total hemoglobin to which glucose is bound. It is therefore a diagnostic tool, as well as a critical endpoint for measuring the progression of T2D, reflecting an individual’s chronic glycemic control over 3 months [23]. This parameter is considered superior to fasting plasma glucose as it exhibits minimal biological variability. Values above 6.5% indicate T2D, and values between 5.7 and 6.4% indicate prediabetes [24]. Regarding HbA1c, the recorded values included percentage levels at baseline and post-intervention. For physical activity data, we included self-reported and objectively measured information. Self-reported data were obtained through standardized questionnaires, where individuals estimated their physical activity over a certain period of time. Objectively measured data were collected using accelerometers or pedometers, which provided direct measurements of physical activity by quantifying activity patterns. Specifically, we gathered information on every physical activity outcome provided, such as total minutes of physical activity per day or week and minutes of moderate-to-vigorous physical activity per day, at baseline and post-intervention. In cases of missing information for conducting the meta-analysis, we proactively contacted the corresponding authors and requested them to provide the missing data.

### Quality Assessment

All studies were qualitatively assessed using the Risk of Bias Assessment Tool [25]. This tool is designed to evaluate the potential sources of bias in RCTs, encompassing various factors that could introduce bias. It assesses multiple aspects related to the randomization process, potential deviations from the planned intervention, handling of missing data, and the measurement of outcomes. Blinding of both participants and study staff when investigating lifestyle interventions was not feasible, as suggested by previous systematic reviews [26, 27], so the evaluation of blinding was excluded. Any disagreements that arose during the assessment process were thoroughly discussed between VH and CZ to reach a consensus.

### Qualitative and Quantitative Data Analysis

We conducted a comprehensive analysis of studies focusing on lifestyle interventions to promote physical activity in patients with T2D. The analysis involved both qualitative and quantitative synthesis of the data. Only studies that provided information to calculate effect sizes were included in the multivariate meta-analyses. We calculated standardized mean differences (Hedges’ *g*) with 95% CIs for physical activity and its different outcomes of subjectively and objectively measured physical activity considering both the pre-test and post-test measurements. Hedges’ g was interpreted as negligible (< 0.2), small (< 0.5), moderate (< 0.8), or large (> 0.8) [28]. In addition to the 95% CI, we also report the prediction interval, as it captures the range of possible treatment effects across diverse settings, offering insight into the expected outcomes for future studies [29]. When assessing the outcome HbA1c, we analyzed the absolute values because they are reported in the same unit of measurement. We computed the difference in the target outcome between the intervention group and the control group for each study arm. Given the observed heterogeneity among the included trials, we utilized a random-effects meta-analysis approach. This model provides a more conservative effect estimate and assumes that the true effect may vary across studies rather than presuming a single true effect size. The random-effects approach accounts for within-study variance and between-study variance, which we felt appropriate given the diversity of interventions studied [30]. The data analysis for changes in physical activity and HbA1c was conducted using the statistical software R.

To address the potential bias caused by having multiple intervention arms and physical activity outcomes in these studies, we followed established instructions to ensure appropriate control [28]. For this purpose, we divided each group’s sample size by the number of comparisons it was part of. We evaluated the heterogeneity of the included studies using *I*^2^, where values above 50% indicate substantial heterogeneity. In order to identify a possible bias in the studies, we created funnel plots. Moreover, we conducted a sensitivity analysis by systematically excluding individual studies and comparing the resultant effects. This analysis aimed to assess the robustness of the results, helping us ascertain the reliability and consistency of our findings. To ensure the reliability of the results, sensitivity analyses were performed, which involved excluding identified outlier studies. This process aimed to improve the overall robustness and accuracy of the findings.

Furthermore, we performed a subgroup analysis differentiating between self-reported and objectively measured physical activity outcomes. We investigated whether the inclusion of healthy diet as a behavioral goal has an effect on the reduction of HbA1c in a multivariate meta-regression analysis. Additionally, we performed a multivariate meta-regression analysis to explore the impact of the moderator variable, physical activity, on the study effect sizes of HbA1c. This analytical approach allowed us to investigate the potential influence of physical activity on the observed outcomes related to HbA1c across the studies in our analysis.

## Results

### Summary of Systematic Literature Search Results

**Fig. 1:**
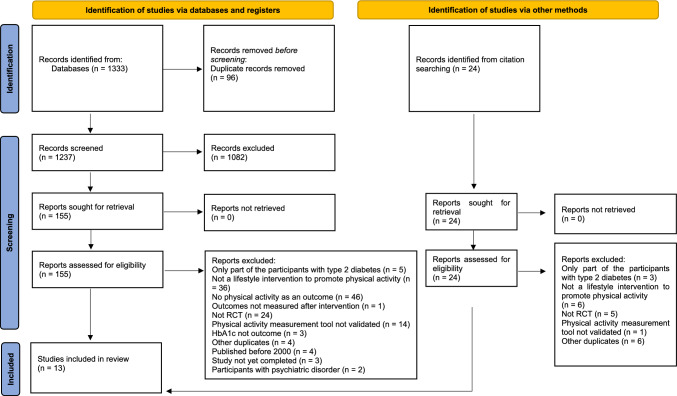
Preferred Reporting Items for Systematic Reviews and Meta-Analyses (PRISMA) flowchart of included and excluded studies. *HbA1c* glycated hemoglobin, *RCT* randomized controlled trial

The study search and selection process is summarized in the PRISMA flowchart (Fig. 1). Initially, 1333 studies were obtained from the literature search in the selected databases, and an additional 24 results were identified through a manual search. After eliminating duplicate studies, there were 1237 remaining studies, which were then screened based on their titles and abstracts. From this screening, 155 studies were deemed eligible for a full-text evaluation, and ultimately, 13 (*n* = 5301 participants) of these studies were included in the review (Table 1).

**Table 1:** Summary of lifestyle interventions to promote physical activity in patients with type 2 diabetes

Study	RCT arms	Baseline sample (*N*)	Baseline n (CG)	Baseline n (IG)	Age, mean (SD)	Women (%)	Control group	PA measurement	Intervention group	Post-measurement (month)	Behavioral goal^e^	Theory of behavioral change	Risk of bias
Andrews et al. (2011) [31] England	3	593	99	246	60	35.1	TAU	Accelerometer (steps/day)	One-on-one meetings, print educational materials, PA^a^ diary	12	PA, healthy diet	Goal-orientated motivational interviews	Low risk
Bender et al. (2017) [32] USA	2	45	23	22	57.6 (9.8)	62.2	Waiting list	Pedometer (steps/day)	App, accelerometer, Facebook group, one–one meeting	6	PA, healthy diet	Social cognitive theory, trans theoretical model for health behavioral change	Low risk
De Greef et al. (2010) [33] Belgium	2	41	21	20	61.3 (6.5)	31.7	TAU	Accelerometer (MVPA min/day, total PA min/day)	Group meetings, pedometer	3	PA	Cognitive-behavioral therapy, motivational interviewing	Low risk
De Greef et al. (2010) [34] IG 1 Belgium	3	67	24	21	67.4 (9.3)	29.9	TAU	Pedometer (steps/day) IPAQ (MVPA min/day, total PA min/day)	Group meetings, pedometer	3	PA	Cognitive-behavioral therapy, motivational interviewing	Some concerns
De Greef et al. (2010) [34] IG 2 Belgium	3	67	24	22	67.4 (9.3)	29.9	TAU	Pedometer (steps/day) IPAQ (MVPA min/day, total PA min/day)	One-on-one meetings, pedometer	3	PA	Cognitive-behavioral therapy, motivational interviewing	Some concerns
Eakin et al. (2013) [35] Australia	2	302	151	151	58 (8.6)	43.71	TAU	Accelerometer (total PA min/week)	Telephone meetings, pedometer	6	PA, healthy diet	Motivational interviewing, social cognitive theory	Some concerns
Glasgow et al. (2012) [36] IG1 USA	3	463	132	169	58.4 (9.2)	49.8	TAU	CHAMPS (weekly caloric expenditure in PA)	Web-based intervention	4	PA, healthy diet, self-management in medication intake	Social cognitive theory, social-ecological model, 5 As self- management model	High risk
Glasgow et al. (2012) [36] IG2 USA	3	463	132	162	58.4 (9.2)	49.8	TAU	CHAMPS (weekly caloric expenditure in PA)	Web-based intervention, automatic computer-based phone calls, telephone meetings, group sessions	4	PA, healthy diet, self-management in medication intake	Social cognitive theory, social-ecological model, 5 As self-management model	High risk
Hansel et al. (2017) [37] France	2	120	60	60	57 (9)	66.7	TAU	IPAQ short (kcals/day)	Web-based intervention	4	PA, healthy diet	None	High risk
Höchsmann et al. (2019) [38] Switzerland	2	36	18	18	–	47.2	Minimal intervention	Accelerometer (steps/day)	Smartphone game	6	PA	None	Some concerns
LookAHEAD study group (2013, 2022) [39, 40]	2	1971	990	981	58.6 (6.8)	59.5	Minimal intervention	Accelerometer (MET minutes per 100) Paffenbarger Questionnaire (MET minutes per 100)	One-on-one meetings, group sessions, telephone meetings, e-mail contact	48	PA, healthy diet	None	Low risk
Lynch et al. (2019) [41] USA	2	211	105	106	55 (10.3)	70.1	Minimal intervention	Accelerometer (steps/day, moderate PA min/week)	Group meetings, telephone meetings	12	Healthy diet, PA, self-management in medication	None	Low risk
Plotnikoff et al. (2013) [42] IG 1 Canada	3	287	94	97	61.6	46	Minimal intervention	Pedometer (steps/3 days) Godin Leisure Time Exercise Questionnaire (MVPA min/week)	Print educational materials, pedometer, PA diary, calendar	12	PA	Integrated stage model	Some concerns
Plotnikoff et al. (2013) [42] IG 2 Canada	3	287	94	96	61.6	46	Minimal intervention	Pedometer (steps/3 days) Godin Leisure Time Exercise Questionnaire (MVPA min/week)	Print educational materials, pedometer, PA diary, calendar, telephone meetings	12	PA	Integrated stage model, motivational interviewing, social cognitive theory	Some concerns
Samuel-Hodge et al. [43] (2009) USA	2	201	84	117	59	63.7	Minimal intervention	Accelerometer (moderate PA min/day, light PA min/day, total PA h/day)	One-on-one meetings, group meetings, telephone meetings, print educational materials	12	PA, healthy diet, self-management	Social cognitive theory, stage of change model, adult learning theory	Low risk
Taheri et al. [44] (2020) Qatar	2	147	77	70	42.1 (5.6)	27.2	TAU	IPAQ (MET min/week)	One-on-one meetings, app, accelerometer	12	Healthy diet, PA	None	Some concerns

*CG* control group, *CHAMPs* Community Healthy Activities Model Program for Seniors, *IG* intervention group, *IPAQ* International Physical Activity Questionnaire, *MET* metabolic equivalent of task, *MVPA* moderate-to-vigorous physical activity, *PA* physical activity, *RCTs* randomized controlled trials, *SD* standard deviation, *TAU* treatment as usual

^a^The main behavioral goal of the intervention is stated first

### Results of the Qualitative Analyses

#### Design of the Studies

We encompassed a total of 13 RCTs within our qualitative analysis [31–38, 41–44]. These trials investigated 16 different lifestyle interventions aimed at promoting physical activity in individuals diagnosed with T2D. Of these 13 RCTs, nine [32, 33, 35, 37–39, 41, 43, 44] were structured as two-arm RCTs, while four [31, 34, 36, 42] were designed as three-arm RCTs. Lifestyle interventions were compared with three types of control groups: treatment as usual group in seven studies [31, 33–37, 44], waiting list control group in one study [32], or minimal intervention in five studies [38, 39, 41–43]. On average, the participants’ mean age was 55 (standard deviation = 5.8) years. As the study by Höchsmann et al. [38] only stated the median including the interquartile range, the study was not included in the calculation of age. Women comprised 50% of the total participants. In most of the studies, the specific procedure for measuring HbA1c was not explicitly detailed. According to the Cochrane Risk of Bias Tool, six of the investigated studies exhibited a low risk of bias [31–33, 41, 43], five studies displayed certain concerns relating to the evaluation of bias [33, 35, 38, 39, 42, 44], and two studies demonstrated a high risk of bias [36, 37] (see ESM).

#### Intervention Components and Intensity

Based on the inclusion criteria of the target group, all studies addressed patients with T2D, but in different contexts. Overall, four studies addressed adults with T2D in a specific cultural background (e.g., Filipino American or Afro-American) [32, 36, 41, 43] and similarly five patients with T2D with overweight [32, 35, 37–39]. Furthermore, two of the studies examined patients in early stage T2D [31, 44], one study patients with manifested T2D, and another study patients with uncontrolled T2D [41].

Three of 13 studies examined more than one lifestyle intervention to promote physical activity. Therefore, 16 interventions were considered in more detail in a further qualitative analysis. The intervention components can be grouped into overarching categories. This categorization provides a clear understanding of how these intervention components were distributed among the analyzed interventions. In the analysis of 15 interventions, therapist-guided components, characterized by a personal contact with the coach, therapist, or trainer, were the most prevalent (12/15) [31–36, 39, 41–44]. Digital components were incorporated into six [32, 36–38, 44] of the interventions. Monitoring and action planning components were incorporated into nine of the reviewed interventions [31–35, 42, 44]. Information-based components were integrated in four [32, 36, 44] interventions.

The length of the intervention averaged 10 months with a range of 3–48 months. In the therapist-guided interventions, characterized by a personal contact, the duration of the intervention per month was identified. Of the 12 publications that examined therapist-guided interventions, eight reported the duration of the therapist-guided components [31, 33, 34, 36, 39, 42, 43], lasting on average 86 min per month with a range of 10–180 min per month.

Overall, all interventions addressed the behavioral goal of promoting physical activity, as this was an inclusion criterion of the review. Of these, ten additionally addressed the goal of promoting healthy eating [31, 32, 35–37, 39, 41, 43, 44] and four addressed self-management in patients with T2D [36, 41, 43].

Out of the 16 interventions investigated, 12 were grounded in distinct national guidelines for T2D or guidelines for groups with specific cultural backgrounds as the foundational framework for their intervention strategies [31–35, 38, 39, 41, 42, 44]. When examining theories, models, and guides for behavioral change, the following could be identified: motivational interviewing [45, 46] (6/15) [31, 33–35, 42], social cognitive theory [47] (6/15) [32, 35, 36, 42, 43], transtheoretical model for health behavioral change [48] (1/15) [32], cognitive-behavioral therapy (3/15) [33, 34, 49], social-ecological model [50] (2/15) [36], 5 “As” self-management model [51] (2/15) [36], integrated stage model [52] (2/15) [42], stages of change model [53] (1/15) [43] and adult learning theory [54] (1/15) [43]. On average, 1.6 (range 0–3) behavioral change theories, models, and/or guidelines were implemented per intervention, with five interventions implementing no theories, models, or guidelines.

### Results of the Quantitative Analyses

#### Effects of Lifestyle Interventions on Physical Activity

**Fig. 2:**
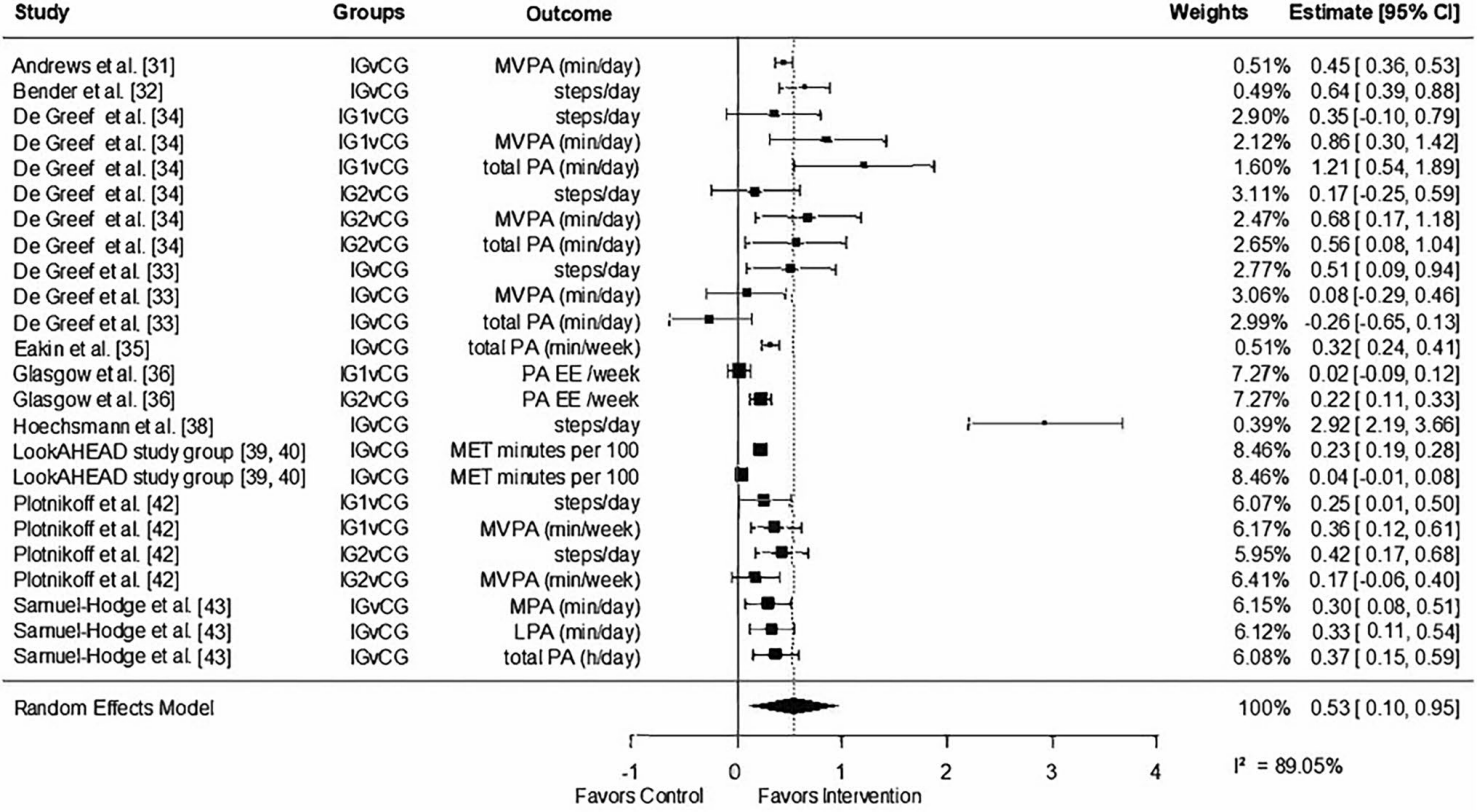
Forest plot random-effect models for the effect size of physical activity. *CG* control, *CI* confidence intervals, *EE* energy expenditure, *h* hours, *IG* intervention group, *IG1* intervention group 1, *IG2* intervention group 2, *LPA* light physical activity, *min* minutes, *MVPA* moderate-to-vigorous physical activity, *PA* physical activity, *v* versus

All data for the quantitative analysis, including sample size and standard deviation or standard error, can be found in the ESM. A total of three studies had to be excluded from the meta-analysis because of unavailable data required for the analysis [37, 41, 44]. Based on the collective physical activity outcome data (*n* = 24) of the interventions, it was observed that lifestyle interventions led to an increase in physical activity, as indicated by an effect size of *g* = 0.53 (95% CI 0.10, 0.96, *I*^2^ = 89.05%) [Fig. 2], in comparison to control groups. A sensitivity analysis, which excluded the study conducted by Höchsmann et al., [38] yielded a reduced effect size of *g* = 0.31 (95% CI 0.19, 0.42, *I*^2^ = 85.99%), but smaller heterogeneity in the studies (refer to the ESM). When examining various physical activity outcomes, the effects were similar for total physical activity (*g* = 0.32, 95% CI − 0.07, 0.72) and moderate-to-vigorous physical activity (*g* = 0.38, 95% CI 0.17, 0.60). Lifestyle interventions had a moderate effect on the outcome steps per day (*g* = 0.43, 95% CI 0.25, 0.61). With an effect size of g = 0.33, this translates to an increase of 9.0 min (95% CI 5.8, 12.2) in total physical activity, 1.7 min (95% CI 1.1, 2.3) in moderate-to-vigorous physical activity, and 126 more steps per day (95% CI 81, 171). We predict that the upcoming measurement of total physical activity, based on prediction intervals, will fall within the range of 0.6–17.5 min per day. For moderate-to-vigorous activity, the expected range is 0.1–3.3 min per day, and for steps, the anticipated range is between 8 and 244 steps per day.

When comparing subjectively measured (*g* = 0.33, 95% CI 0.06, 0.59, *I*^2^ = 77.07%) and objectively measured (*g* = 0.30, 95% CI 0.17, 0.43, *I*^2^ = 89.25%) physical activity, no relevant differences emerged after exclusion of the study conducted by Höchsmann et al. [38] (refer to the ESM). However, when including this particular study, we found a higher effect size of the studies that used objective instruments to measure physical activity (*g* = 0.54, 95% CI 0.05, 1.04, *I*^2^ = 91.97%) [refer to the ESM]).

**Fig. 3:**
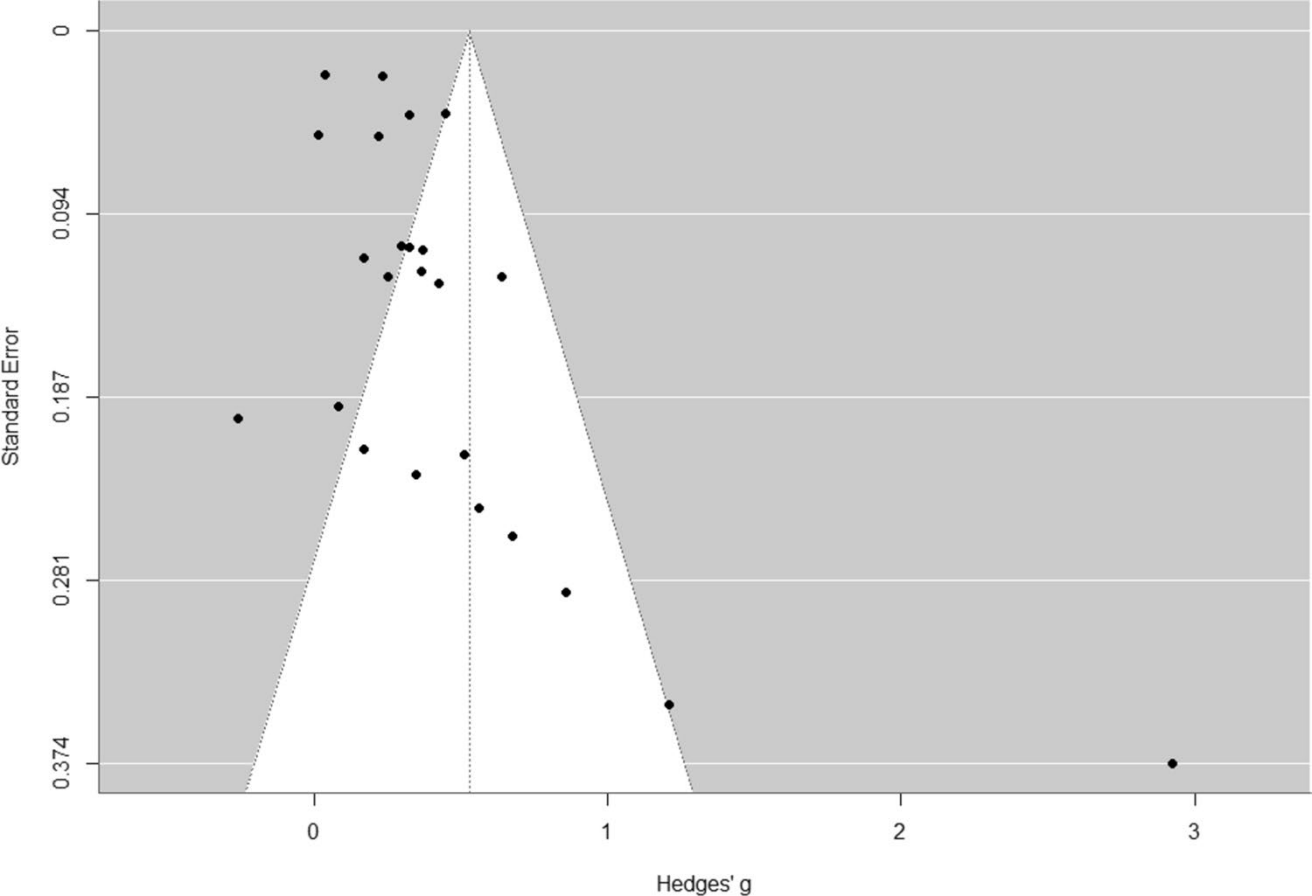
Funnel plot to identify bias for the outcome physical activity

In summary, the funnel plot (Fig. 3) depicting the outcome of physical activity exhibits asymmetry, potentially suggesting underlying factors such as publication bias, heterogeneity among the studies, or methodological discrepancies [55]. It is possible that studies with small sample sizes have not been published. There was a single study displaying a high effect size of g = 2.92 (95% CI 2.19, 3.66), coupled with a substantial standard error [38]. This particular study may have exerted an impact on the overall effect size of the meta-analysis, prompting the consideration of a sensitivity analysis. The relevance of the sensitivity analysis is further underscored by the uniqueness of this study, as it is the sole study examining the use of a smartphone game as a means to promote physical activity. The primary objective of the smartphone game was to encourage individuals to enhance their daily physical activity by increasing the number of steps taken. The app measured daily steps through the phone’s sensors, and users were rewarded for both their daily step count and their participation in the app’s internal training. These rewards were intricately tied to progressing within the game. Consequently, it is not surprising that participants were motivated to boost their daily step count, aligning with the explicit goal of the app. Post-measurements occurred during the first week following the 24-week intervention period. It is possible that some participants might have continued playing the game after this period. After the intervention ended, study participants were allowed to continue using the game; however, it was not evaluated whether they actually did so.

#### Effects of Lifestyle Interventions on HbA1c

**Fig. 4:**
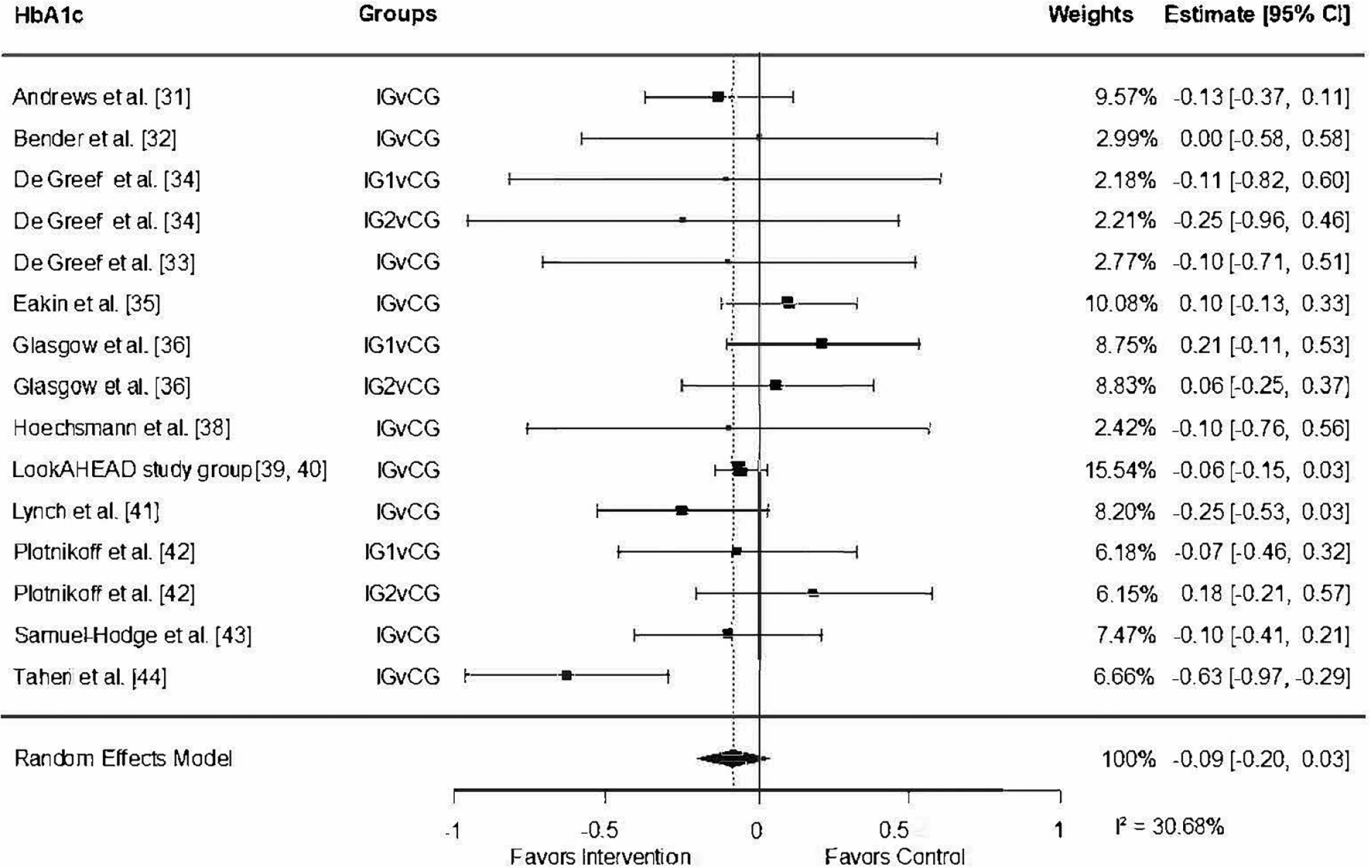
Forest plot random-effect models for the outcome glycated hemoglobin. *CG* control, *CI* confidence interval, *IG* intervention group, *IG1* intervention group 1, *IG2* intervention group 2, *v* versus

**Fig. 5:**
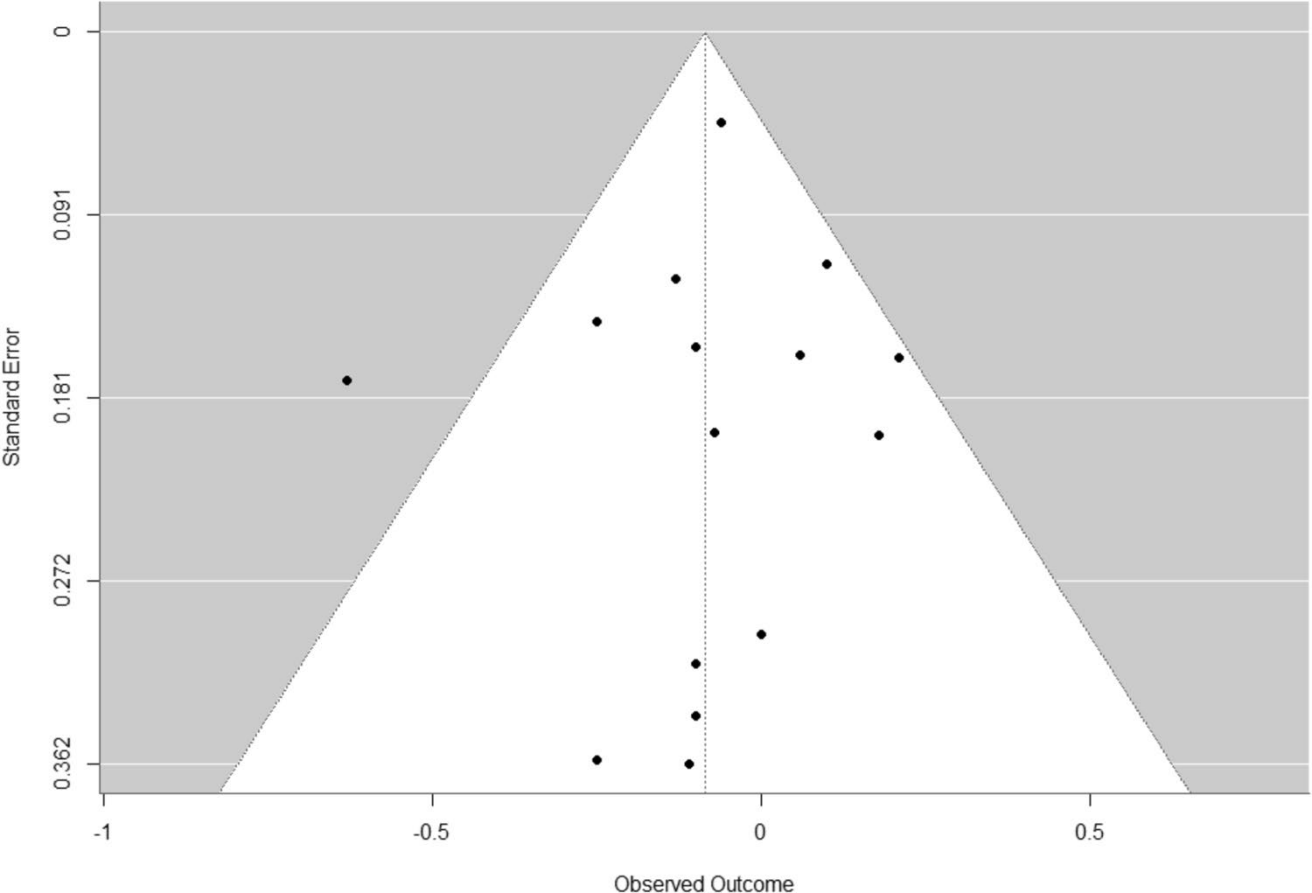
Funnel plot to identify bias for the glycated hemoglobin outcome

With an initial mean of 7.40% in HbA1c at baseline, there was a reduction of 0.09% (95% CI − 0.20, 0.03, *I*^2^ = 30.68%) of HbA1c, measured from baseline to post-measurement in the intervention groups (Fig. 4). The funnel plot illustrating the outcome of HbA1c displayed variability in the effects and standard errors among the included studies. However, two studies stood out from this overall effect size, suggesting the presence of potential heterogeneity or methodological disparities among the studies (Fig. 5). One of these studies, which reported the highest effect on HbA1c (*g* = − 0.50, 95% CI − 0.61, − 0.35), introduced a distinctive phase at the beginning of the intervention involving a comprehensive shift to a low-energy diet [44]. This substantial dietary modification could account for the markedly higher effect observed in this particular study. The second study that stood out represented a negative effect but showed no methodological anomalies apart from some concerns about the study quality [42].

A moderator analysis revealed that studies targeting a healthy diet as a behavioral goal were associated with a reduction in HbA1c of − 0.04 (95% CI − 0.29, 0.22). This result indicates that addressing a healthy diet as a behavioral goal does not moderate the impact of lifestyle interventions on HbA1c levels.

#### Physical Activity as a Moderator of the Impact of Lifestyle Interventions on HbA1c

A multivariate meta-regression was conducted to explore whether physical activity plays a moderating role in determining the effects of lifestyle interventions on the outcome HbA1c. A total of *n* = 24 intervention outcomes related to physical activity (15 objectively measured, 9 subjectively measured) and HbA1c were included in the analysis. Higher physical activity was associated with a decrease of − 0.04 (95% CI − 0.15, 0.06) in HbA1c. These findings indicate that physical activity does not moderate the impact of lifestyle interventions on HbA1c effect.

### Study Quality and Effects of Lifestyle Interventions on Physical Activity and HbA1c

Regarding the meta-regression analyzing the impact of study quality on physical activity, there appeared to be a slightly higher overall effect observed in studies of high quality, on average. Studies with higher study quality had an effect size of *g* = 0.41 (95% CI − 0.47, 1.03) greater than studies of lower quality. However, it is essential to highlight that this observed effect was primarily influenced by the study by Höchsmann et al. [38]. When this specific study was excluded from the analysis, the previously observed association disappeared, rendering the differences trivial.

The meta-regression revealed a generally minuscule lower average effect in high-quality studies when HbA1c was used as an outcome (*g* = 0.02, 95% CI − 0.22, 0.26). This implies that the observed difference in effects between high-quality and low-quality studies was not relevant.

## Discussion

This systematic review and meta-analysis aimed to summarize RCTs on lifestyle interventions and to assess their impact on physical activity and HbA1c. Overall, there was a limited number of studies investigating lifestyle interventions for T2D. These interventions exhibit considerable heterogeneity concerning their design, components, and duration. A variety of interventions involve direct interaction with a therapist, trainer, or coach, or incorporate a digital component. While the promotion of physical activity is a focus, more than half of the interventions also addressed the behavioral objective of fostering a healthy diet. The predominant theories guiding behavioral change in these studies have mostly a social cognitive basis, although other models and guidelines on behavior modification were also utilized. Overall, the examined studies demonstrated a moderate effect, with a subsequent sensitivity analysis revealing a small effect of enhancing physical activity. Regarding HbA1c, lifestyle interventions showed no relevant effect compared to control groups. The studies analyzed did not indicate that physical activity acts as a moderator of the interventions on HbA1c.

### Effect of Lifestyle Interventions on Physical Activity

Our meta-analysis revealed a small effect of increasing physical activity through lifestyle interventions when compared with a control group. If an individual were to increase their daily total physical activity by 9.0 min, this change would potentially lead to an estimated reduction of 18.8% in their risk of mortality when compared with the baseline. This is a proportional estimate derived from the initial data suggesting that 23 min per day is associated with a 48% reduction in mortality [56]. The small effect on physical activity aligns with the collective findings of 18 meta-analyses focused on lifestyle interventions, specifically targeting physical activity outcomes in different populations (*d* = 0.26, 95% CI 0.21, 0.31) [57]. One observation in particular emerged from this meta-analysis: a predominant focus of interventions on theories of behavioral change rooted in the fundamental principles of cognitivism. This sole focus is critical as researchers have started to emphasize the crucial role of affective and emotional responses to physical activity for motivated behavior and sustained physical activity [58]. Additionally, it seems that the primary motivators for engaging in physical activity revolve around the enjoyment of the experience and overall well-being [58, 59]. In addition to the reasons listed above, this might be one explanation why the intervention conducted by Höchsmann et al. [38] demonstrated an exceptionally high effect size. The incorporation of manageable challenges in the intervention could be perceived as especially enjoyable, thereby boosting motivation. Similar findings regarding the effectiveness are evident in other studies, indicating that mobile apps that integrate gaming with physical activity result in increases in physical activity [60]. Giving due attention to positive emotional responses, such as pleasure and joy, as well as acknowledging the role of core affective responses, can contribute to fostering lifelong motivation for physical activity and exercise [61]. A combination of interventions that blend informational and experiential approaches, considering training-related effects, may prove effective in promoting physical activity in patients with T2D.

### Effect of Lifestyle Interventions on HbA1c

Our meta-analysis for the outcome HbA1c showed that lifestyle interventions to promote physical activity had a negligible effect on HbA1c compared to the control group over an average period of 7 months. It is possible that the promotion of physical activity by lifestyle interventions studied was not sufficient to affect HbA1c. Structured exercise interventions exhibited a capacity for positive effects on HbA1c progression. The examination of meta-analyses focusing on structured exercise programs and their impact on HbA1c reduction revealed mean differences that ranged between − 0.16 and − 0.71% [18–21]. Unsupervised exercise training did not manifest substantial effects [20]. Exercise modes, including aerobic exercise, strength exercise, and their combination, demonstrated clear but modest reductions in HbA1c [11]. Nevertheless, adherence to exercise programs tends to be low, unless they are coupled with a psychological component [12]. Further investigation is needed to address the question of whether individuals with T2D should receive structured exercise programs, known for their positive impact on HbA1c, as an alternative to lifestyle interventions. Additionally, there is a necessity to explore the extent to which lifestyle interventions can be enhanced to improve their efficacy in influencing HbA1c. Here, it is essential to recognize the disparity between the efficacy observed in exercise interventions and the practical effectiveness evident in lifestyle interventions.

The lifestyle interventions examined in this meta-analysis varied in their incorporation of a healthy diet component, a factor that could potentially account for the observed absence of relevant effects on HbA1c. Evidence suggests that the combination of dietary modifications and physical activity has a beneficial impact, either reducing or delaying the onset of T2D [62, 63]. Considering this, it is plausible that improvement of a healthy diet may enhance the efficacy of lifestyle interventions in influencing HbA1c. A sensitivity analysis was performed on the HbA1c outcome, comparing studies that included a healthy diet as a behavioral goal in the intervention with those that did not. No meaningful difference was observed, suggesting that solely addressing nutrition as a behavioral goal may not be adequate to impact HbA1c. Rather than investigating the incorporation of the behavioral goal of a healthy diet, it is reasonable to explore the influence of lifestyle interventions on diet as an additional outcome. This broader perspective is essential for a comprehensive understanding of how lifestyle interventions affect HbA1c.

Additionally, the influence of treatment as usual should be considered, as participants in the control group may continue to receive medication for T2D. This could potentially clarify the absence of group differences in this outcome. To address this issue, one approach is to establish a predetermined target for initiating medication and ensure that the intervention allocation is blinded to the physician [31]. However, this may not always be feasible because of ethical considerations and may not align with studies conducted in pragmatic settings [64]. Nevertheless, it is essential to consider medication as an outcome in the studies. The efficacy of lifestyle interventions might not be evident in the reduction of HbA1c alone but could manifest in the diminished reliance on medication.

Our meta-regression contradicted the hypothesis that increased physical activity moderates the HbA1c value. As previously discussed, factors such as the lack of consideration for implementing a healthy diet might contribute to this result. Additionally, the diverse range of intervention approaches utilized in the studies, including variations in duration, components, integrated elements, and underlying theories and guidelines, could also influence the results. It is plausible that inadequate participant engagement or inconsistent adherence to the interventions may further complicate the identification of a moderating effect.

### Strengths and Limitations

This systematic review and meta-analysis provide a comprehensive evaluation of the effects of lifestyle interventions on both physical activity and HbA1c in patients with T2D, which is essential for understanding the full impact of these interventions on T2D management. By including studies that report both outcomes, we ensure results that align with existing guidelines. The American Diabetes Association emphasizes the importance of addressing physical activity and clinical parameters such as HbA1c to improve diabetes management [65]. The systematic review offers a thorough examination of the structural components and objectives of the interventions, providing insights into the study designs. The review stands out by focusing on interventions that go beyond exercise prescription, emphasizing the incorporation of physical activity into patients’ daily lives. A further strength lies in its incorporation of current findings from theoretical sport and health-related psychology, adding depth to the discussion and elucidating implications for enhancing intervention effectiveness. In conclusion, this systematic review, coupled with a meta-analysis, constitutes a valuable addition to the landscape of high-quality reviews on physical activity and exercise in individuals with T2D [11, 66–69].

However, this study also has certain limitations that need clarification. Although the term “lifestyle intervention” is clearly defined [14], its consistent usage across various studies and reviews remains a concern, often lacking a clear differentiation from exercise interventions [70]. Moreover, the appropriateness of HbA1c as the sole outcome measure for assessing the effectiveness of lifestyle interventions is debatable. Although HbA1c is a suitable metric for tracking the progression of T2D, the pragmatic nature of lifestyle interventions in real-world settings raises questions. Participants in the control group, unable to effectively manage their T2D through lifestyle modifications, may require increased medication to control T2D. To address this, it is suggested that additional outcomes, such as medication usage and body mass index, be considered for a more comprehensive evaluation of intervention effectiveness. This approach would provide a more nuanced understanding of the multi-faceted outcomes associated with lifestyle interventions in the context of T2D management. An additional limitation of this review is the potential variability introduced by medical adjustments, such as medication changes within some lifestyle intervention studies, which could have influenced the observed HbA1c outcomes and complicated interpretation of intervention effects.

## Conclusions and Outlook

Despite the success of studied lifestyle interventions in promoting physical activity, our review found that their overall effectiveness proved to be small after a sensitivity analysis. While the prevalent focus on cognitivism in behavioral change theories was evident in the examined interventions, there is a recognized potential for enhancing motivation for physical activity in individuals with T2D by acknowledging emotional responses, pleasure, and overall well-being. The effectiveness of promoting sustained physical activity may be further increased through a strategic combination of informational and experiential approaches in exercise.

The observed lack of a relevant impact on HbA1c in comparison to control conditions raises questions about the effectiveness of the studied lifestyle interventions in achieving meaningful improvements in glycemic control among individuals with T2D. This emphasizes the importance of exploring alternative strategies that could better address glucose regulation in this population. Given the well-established positive impact of structured exercise programs on HbA1c, there is a possibility that these programs might offer a more promising approach than lifestyle interventions for individuals with T2D. However, adherence to participation in exercise interventions is rather low. On the one hand, it is necessary to explore enhancements in lifestyle interventions to more effectively influence HbA1c. On the other hand, attention should be given to improving adherence to structured exercise programs, which have demonstrated effectiveness in impacting HbA1c. The inconclusive role of physical activity as a moderator signals the need for a better understanding of how changes in physical activity may directly contribute to the effectiveness of these interventions in managing HbA1c. Furthermore, it underscores the disparity between the observed efficacy of exercise interventions and the practical effectiveness seen in lifestyle interventions.

## Supplementary Information

Below is the link to the electronic supplementary material.

Supplementary file1 (PDF 169 KB)

Supplementary file2 (PDF 229 KB)

Supplementary file3 (PDF 214 KB)

## Declarations

### Availability of data and material

Data are available in the ESM.
