# Supplementary material for: Effects of Lifestyle Interventions to Promote Physical Activity on Physical Activity and Glycated Hemoglobin in Patients with Type 2 Diabetes: a Systematic Review and Meta-Analysis
Source: Sports Med. 2025 Mar 13;55(5):1165–81. doi: 10.1007/s40279-025-02184-8 (PMC12106156; doi:10.1007/s40279-025-02184-8)
Supplement: Supplementary file 1 — Supplementary file1 (PDF 169 KB) [file 40279_2025_2184_MOESM1_ESM.pdf]

Effects of lifestyle interventions to promote physical activity on glycated hemoglobin and physical activity in patients with T2D - Systematic review and meta-analysis

Sports Medicine

Vivien Hohberg [1]

Eric Lichtenstein [1]

Jan-Niklas Kreppke [1]

Cedrine Zanitti [1]

Fiona Streckmann [1]

Markus Gerber [1]

Oliver Faude [1]

1) Department of Sport, Exercise and Health, University of Basel, Switzerland

Study quality

Study ID	D1	D2	D3	D4	D5	Overall
Andrews et al. (2011)	+	+	+	+	+	+
Bender et al. (2017)	+	+	+	+	+	+
De Greef et al. (2010a)	+	+	+	+	+	+
De Greef et al. (2010b)	+	+	+	!	+	!
Eakin et al. (2013)	!	+	+	+	+	!
Glasgow et al. (2010)	!	+	+	-	+	-
Hansel et al. (2017)	+	+	+	-	!	-
Hoechsmann et al. (2019)	+	+	+	-	+	!
Lynch et al. (2019)	+	+	+	+	+	+
Plotnikoff et al. (2013)	+	+	+	!	+	!
Samuel-Hodge et al. (2009)	+	+	+	+	+	+
Taheri et al. (2020)	+	+	+	-	+	!
LookAHEAD study group (2013, 2022)	+	+	+	+	+	+

+	Low risk
!	Some concerns
-	High risk

D1	Randomisation process
D2	Deviations from the intended interventions
D3	Missing outcome data
D4	Measurement of the outcome
D5	Selection of the reported result
