# Supplementary material for: Effects of Lifestyle Interventions to Promote Physical Activity on Physical Activity and Glycated Hemoglobin in Patients with Type 2 Diabetes: a Systematic Review and Meta-Analysis
Source: Sports Med. 2025 Mar 13;55(5):1165–81. doi: 10.1007/s40279-025-02184-8 (PMC12106156; doi:10.1007/s40279-025-02184-8)
Supplement: Supplementary file 2 — Supplementary file2 (PDF 229 KB) [file 40279_2025_2184_MOESM2_ESM.pdf]

Effects of lifestyle interventions to promote physical activity on glycated hemoglobin and physical activity in patients with T2D - Systematic review and meta-analysis

Sports Medicine

Vivien Hohberg [1]

Eric Lichtenstein [1]

Jan-Niklas Kreppke [1]

Cedrine Zanitti [1]

Fiona Streckmann [1]

Markus Gerber [1]

Oliver Faude [1]

1) Department of Sports, Exercise and Health, University of Basel, Switzerland

Data for meta analysis: Outcome physical activity

Authors	outcome	IG_vs_CG	intervention_duration	study_quality	Measurement_method	Number_of_RCT_arms	Control_condition	n_IG	mean_pre_IG	SD_pre_IG	SE_pre_IG	mean_post_IG	SD_post_IG	SE_post_IG	n_CG	MW_pre_CG	SD_pre_CG	SE_pre_CG	mean_post_CG	SD_post_CG	SE_post_CG
Andrews et al. (2011)	MVPA (min/day)	IGvCG	12	low	Accelerometer (MVPA, min/day)	3	TAU	240	23	17		31	25		93	26	20		26	23	
Bender et al. (2017)	steps/day	IGvCG	6	low	Accelerometer (steps/day)	2	Waitinglist	22	7483	2415		9524	3626		23	6735	2363		7208	2719	
De Greef et al. (2010a)	steps/day	IGvCG	3	low	linear Pedometer (steps/day)	2	TAU	6.7	7099	4208		9601	5002		7	5214	3352		5538	3877	
De Greef et al. (2010a)	MVPA (min/day)	IGvCG	3	low	Accelerometer (MVPA, min/day)	2	TAU	6.7	39	35		44	38		7	23	29		25	29	
De Greef et al. (2010a)	total PA (min/day)	IGvCG	3	low	Accelerometer (total PA, min/day)	2	TAU	6.7	255	87		253	99		7	223	84		246	109	
De Greef et al. (2010b)	steps/day	IG1vCG	3	some_concerns	Pedometer (steps/day)	3	TAU	7	5924	3725		7630	4423		4	4831	2601		5173	3094	
De Greef et al. (2010b)	MVPA (min/day)	IG1vCG	3	some_concerns	IPAQ (MVPA, min/day)	3	TAU	7	46	43		75	64		4	44	52		27	39	
De Greef et al. (2010b)	total PA (min/day)	IG1vCG	3	some_concerns	IPAQ (total PA, min/day)	3	TAU	7	113	74		195	106		4	86	72		65	68	
De Greef et al. (2010b)	steps/day	IG2vCG	3	some_concerns	Pedometer (steps/day)	3	TAU	7.3	5114	2503		5951	3191		4	4831	2601		5173	3094	
De Greef et al. (2010b)	MVPA (min/day)	IG2vCG	3	some_concerns	IPAQ (MVPA, min/day)	3	TAU	7.3	60	60		88	100		4	44	52		27	39	
De Greef et al. (2010b)	total PA (min/day)	IG2vCG	3	some_concerns	IPAQ (total PA, min/day)	3	TAU	7.3	126	86		158	110		4	86	72		65	68	
Eakin et al. (2013)	total PA (min/week)	IGvCG	6	some_concerns	Accelerometer (total PA, min/week)	2	TAU	151	125.2	114.7		164	160.4		151	120.2	113.9		122	109.1	
Glasgow et al. (2012)	PA EE /week	IG1vCG	4	high	CHAMPS (weekly caloric expenditure in PA)	3	TAU	120	4302	2552.3871	233	3307	2760.52169	252	57	3915	2219.65132	294	2882	2264.950331	300
Glasgow et al. (2012)	PA EE /week	IG2vCG	4	high	CHAMPS (weekly caloric expenditure in PA)	3	TAU	124	3662	2561.1716	230	3174	2839.55983	255	57	3915	2219.65132	294	2882	2264.950331	300
Hoechsmann et al. (2019)	steps/day	IGvCG	6	some_concerns	Accelerometer (steps/day)	2	minimal intervention	18	5785	793		9783	1334		17	5612	1192		6552	1280	
LookAHEAD group (2013 & 2022)	MET minutes per 100	IGvCG	48	low	Accelerometer (MET minutes per 100)	2	minimal intervention	492	4.38	6.89		4.51	10.7		497	4.27	7.76		4.12	28.24	
LookAHEAD group (2013 & 2022)	MET minutes per 100	IGvCG	48	low	Questionnaire (MET minutes per 100)	2	minimal intervention	491	5.19	6.53		7.44	9.18		495	5.21	7.15		5.87	7.45	
Plotnikoff et al. (2013)	steps/day	IG1vCG	12	some_concerns	Pedometer (3-day steps total)	3	minimal intervention	20.7	21279	3498.7387	769	20821	4208.49587	925	13	21409	2758.24673	765	20097	2996.21311	831
Plotnikoff et al. (2013)	MVPA (min/week)	IG1vCG	12	some_concerns	Godin Leisur Time Exercise Questionnaire (MVPA, min/week)	3	minimal intervention	23.7	164.1	83.734151	17.2	174.5	97.3652916	20	14	156.7	65.1048385	17.4	137.8	68.47233018	18.3
Plotnikoff et al. (2013)	steps/day	IG2vCG	12	some_concerns	Pedometer (3-day steps total)	3	minimal intervention	22.6	21338	3617.7527	761	21493	4202.48802	884	13	21409	2758.24673	765	20097	2996.21311	831
Plotnikoff et al. (2013)	MVPA (min/week)	IG2vCG	12	some_concerns	Godin Leisur Time Exercise Questionnaire (MVPA, min/week)	3	minimal intervention	24.3	158.9	84.787452	17.2	153.8	359.85372	73	14	156.7	65.1048385	17.4	137.8	68.47233018	18.3
Samuel-Hodge et al. (2009)	MPA (min/day)	IGvCG	12	low	Acti (moderate PA, min/day)	2	minimal intervention	26.7	8.3	5.1672043	1	8.1	5.16720427	1	18.7	7.3	6.05408953	1.4	5.4	2.594609797	0.6
Samuel-Hodge et al. (2009)	LPA (min/day)	IGvCG	12	low	Acti (light PA, min/day)	2	minimal intervention	26.7	52.3	24.28586	4.7	50.6	22.2189784	4.3	18.7	53.8	25.946098	6	43.7	9.08113429	2.1
Samuel-Hodge et al. (2009)	total PA (h/day)	IGvCG	12	low	Accelerometer (total PA, h/day)	2	minimal intervention	26.7	12.1	1.5501613	0.3	11.9	1.03344085	0.2	18.7	12.4	0.86486993	0.2	11.7	0.864869932	0.2

Data for meta analysis: HbA1c

Authors	IG_vs_CG	intervention_duration	study_quality	Number_of_RCT_arms	Control_condition	healthy_eating_component	n_IG	mean_pre_IG	SD_pre_IG	SE_pre_IG	mean_post_IG	SD_post_IG	SE_post_IG	n_CG	MW_pre_CG	SD_pre_CG	SE_pre_CG	mean_post_CG	SD_post_CG	SE_post_CG
Andrews et al. (2011)	IGvCG	12	low	3	TAU	1	240	6.69	0.99		6.65	0.93		93	6.72	1.02		6.81	0.91	
Bender et al. (2017)	IGvCG	6	low	2	Waitinglist	1	22	7.4	0.82		7.1	0.98		23	7.4	0.93		7.1	1.2	
De Greef et al. (2010a)	IGvCG	3	low	2	TAU	0	20	7.5	1.1		7.3	1.1		21	8	1.3		7.9	1.3	
De Greef et al. (2010b)	IG1vCG	3	some_concerns	3	TAU	0	21	7.12	1.35		6.94	1.19		12	7	0.87		6.93	0.84	
De Greef et al. (2010b)	IG2vCG	3	some_concerns	3	TAU	0	22	7.23	0.71		6.91	0.6		12	7	0.87		6.93	0.84	
Eakin et al. (2013)	IGvCG	6	some_concerns	2	TAU	1	151	7.4	1.5		7.5	1.7		151	7.5	1.7		7.5	1.6	
Glasgow et al. (2012)	IG1vCG	4	high	3	TAU	1	120	8.03	1.533623	0.14	8.1	1.5336232	0.14	57	8.16	1.2079735	0.16	8.02	1.0569768	0.14
Glasgow et al. (2012)	IG2vCG	4	high	3	TAU	1	124	8.26	1.447619	0.13	8.2	1.4476187	0.13	57	8.16	1.2079735	0.16	8.04	1.0569768	0.14
Hoechsmann et al. (2019)	IGvCG	6	some_concerns	2	minimal intervention	0	18	6.2	0.6		6.2	0.7		17	6.9	0.7		7	1	
LookAHEAD group (2013 & 2022)	IGvCG	48	low	2	minimal intervention	1	984	7.17	1.08		6.99	1.36		994	7.25	1.15		7.13	1.41	
Lynch et al. (2019)	IGvCG	12	low	2	minimal intervention	1	99	9.1	1.79		8.4	1.87		97	8.88	1.53		8.43	1.54	
Plotnikoff et al. (2013)	IG1vCG	12	some_concerns	3	minimal intervention	0	71	7.08	0.58983	0.07	7	0.674092	0.08	39.5	7.08	0.4399432	0.07	7.07	0.4399432	0.07
Plotnikoff et al. (2013)	IG2vCG	12	some_concerns	3	minimal intervention	0	70	7.11	0.585662	0.07	7.28	0.669328	0.08	39.5	7.08	0.4399432	0.07	7.07	0.4399432	0.07
Samuel-Hodge et al. (2009)	IGvCG	12	low	2	minimal intervention	1	101	7.8	2.009975	0.2	7.5	1.0049876	0.1	69	7.8	2.4919872	0.3	7.6	0.8306624	0.1
Taheri et al. (2020)	IGvCG	12	some_concerns	2	TAU	1	67	6.95	1.4		5.96	0.84		75	6.95	1.22		6.59	0.92	

Data for meta regression: HbA1c and physical activity

Authors	IG_vs_CG	intervention_duration	study_quality	Number_of_RCT_arms	Control_condition	n_IG	mean_pre_IG	SD_pre_IG	SE_pre_IG	mean_post_IG	SD_post_IG	SE_post_IG	n_CG	MW_pre_CG	SD_pre_CG	SE_pre_CG	mean_post_CG	SD_post_CG	SE_post_CG	Measurement_method	PA_n_IG	PA_mean_pre_IG	PA_SD_pre_IG	PA_mean_post_IG	PA_SD_post_IG	PA_n_CG	PA_mean_pre_CG	PA_SD_pre_CG	PA_mean_post_CG	PA_SD_post_CG	hg	hg_f
Andrews et al. (2011)	IGvCG	12	low	3	TAU	240	6.69	0.99		6.65	0.93		93	6.72	1.02		6.81	0.91		Accelerometer (MVPA, min/day)	240	23	17	31	25	93	26	20	26	23	0.445628	0.45
Bender et al. (2017)	IGvCG	6	low	2	Waitinglist	22	7.4	0.82		7.1	0.98		23	7.4	0.93		7.1	1.2		Accelerometer (steps/day) linear	22	7483	2415	9524	3626	23	6735	2363	7208	2719	0.637408	0.64
De Greef et al. (2010a)	IGvCG	3	low	2	TAU	20	7.5	1.1		7.3	1.1		21	8	1.3		7.9	1.3		Pedometer (steps/day)	6.7	7099	4208	9601	5002	7	5214	3352	5538	3877	0.513128	0.53
De Greef et al. (2010a)	IGvCG	3	low	2	TAU	20	7.5	1.1		7.3	1.1		21	8	1.3		7.9	1.3		Accelerometer (MVPA, min/day)	6.7	39	35	44	38	7	23	29	25	29	0.083615	0.09
De Greef et al. (2010a)	IGvCG	3	low	2	TAU	20	7.5	1.1		7.3	1.1		21	8	1.3		7.9	1.3		Accelerometer (total PA, min/day)	6.7	255	87	253	99	7	223	84	246	109	-0.26138	-0.27
De Greef et al. (2010b)	IG1vCG	3	some_concerns	3	TAU	21	7.12	1.35		6.94	1.19		12	7	0.87		6.93	0.84		Pedometer (steps/day)	7	5924	3725	7630	4423	4	4831	2601	5173	3094	0.346631	0.36
De Greef et al. (2010b)	IG1vCG	3	some_concerns	3	TAU	21	7.12	1.35		6.94	1.19		12	7	0.87		6.93	0.84		IPAQ (MVPA, min/day)	7	46	43	75	64	4	44	52	27	39	0.858354	0.89
De Greef et al. (2010b)	IG1vCG	3	some_concerns	3	TAU	21	7.12	1.35		6.94	1.19		12	7	0.87		6.93	0.84		IPAQ (MVPA, min/day)	7	113	74	195	106	4	86	72	65	68	1.210614	1.25
De Greef et al. (2010b)	IG2vCG	3	some_concerns	3	TAU	22	7.23	0.71		6.91	0.6		12	7	0.87		6.93	0.84		Pedometer (steps/day)	7.3	5114	2503	5951	3191	4	4831	2601	5173	3094	0.16918	0.17
De Greef et al. (2010b)	IG2vCG	3	some_concerns	3	TAU	22	7.23	0.71		6.91	0.6		12	7	0.87		6.93	0.84		IPAQ (MVPA, min/day)	7.3	60	60	88	100	4	44	52	27	39	0.677582	0.70
De Greef et al. (2010b)	IG2vCG	3	some_concerns	3	TAU	22	7.23	0.71		6.91	0.6		12	7	0.87		6.93	0.84		IPAQ (total PA, min/day)	7.3	126	86	158	110	4	86	72	65	68	0.56174	0.58
Eakin et al. (2013)	IGvCG	6	some_concerns	2	TAU	151	7.4	1.5		7.5	1.7		151	7.5	1.7		7.5	1.6		Accelerometer (total PA, min/week)	151	125.2	114.7	164	160.4	151	120.2	113.9	122	109.1	0.322359	0.32
Glasgow et al. (2012)	IG1vCG	4	high	3	TAU	120	8.03	1.53362316	0.14	8.1	0.0523832	0.14	57	8.16	1.20797351	0.16	8.02	0.056	0.14	CHAMPS (weekly caloric expenditure in PA)	120	4302	2552.39	3307	3846.61306	57	3915	3139.06101	2882	5143.92846	0.015394	0.01
Glasgow et al. (2012)	IG2vCG	4	high	3	TAU	124	8.26	1.44761873	0.13	8.2	0.04687217	0.13	57	8.16	1.20797351	0.16	8.04	0.056	0.14	CHAMPS (weekly caloric expenditure in PA)	124	3662	2561.17	3174	3867.26648	57	3915	3139.06101	2882	5143.92846	0.220049	0.20
Hoechsmann et al. (2019)	IGvCG	6	some_concerns	2	minimal intervention	18	6.2	0.6		6.2	0.7		17	6.9	0.7		7	1		Accelerometer (steps/day)	18	5785	793	9783	1334	17	5612	1192	6552	1280	2.923616	2.96
LookAHEAD group (2013 & 2022)	IGvCG	48	low	2	minimal intervention	1	984	7.17	1.08		6.99	1.36		994	7.25	1.15		7.13	1.41	Accelerometer (MET minutes per 100)	492	4.38	6.89	4.51	10.7	497	4.27	7.76	4.12	28.24		
LookAHEAD group (2013 & 2022)	IGvCG	48	low	2	minimal intervention	1	984	7.17	1.08		6.99	1.36		994	7.25	1.15		7.13	1.41	Questionnaire (MET minutes per 100)	490.5	5.19	6.53	7.44	9.18	495	5.21	7.15	5.87	7.45		
Plotnikoff et al. (2013)	IG1vCG	12	some_concerns	3	minimal intervention	71	7.08	0.58983048	0.07	7	0.02116601	0.08	39.5	7.08	0.43994318	0.07	7.07	0.01852026	0.07	Pedometer (3-day steps total)	20.7	21279	6055.11	20821	25651.0356	13	21409	6756.29706	20097	22984.3243	0.253329	0.13
Plotnikoff et al. (2013)	IG1vCG	12	some_concerns	3	minimal intervention	71	7.08	0.58983048	0.07	7	0.02116601	0.08	39.5	7.08	0.43994318	0.07	7.07	0.01852026	0.07	Godin Leisur Time Exercise Questionnaire (MVPA, min/week)	23.7	164.1	144.93	174.5	82.9457654	14	156.7	159.473634	137.8	76.3353522	0.364984	0.19
Plotnikoff et al. (2013)	IG2vCG	12	some_concerns	3	minimal intervention	70	7.11	0.58566202	0.07	7.28	0.02116601	0.08	39.5	7.08	0.43994318	0.07	7.07	0.01852026	0.07	Pedometer (3-day steps total)	22.6	21338	6275.37	21493	24386.2259	13	21409	6756.29706	20097	22984.3243	0.423375	0.22
Plotnikoff et al. (2013)	IG2vCG	12	some_concerns	3	minimal intervention	70	7.11	0.58566202	0.07	7.28	0.02116601	0.08	39.5	7.08	0.43994318	0.07	7.07	0.01852026	0.07	Godin Leisur Time Exercise Questionnaire (MVPA, min/week)	24.3	158.9	146.957	153.8	302.752044	14	156.7	159.473634	137.8	76.3353522	0.170165	0.09
Samuel-Hodge et al. (2009)	IGvCG	12	low	2	minimal intervention	101	7.8	2.00997512	0.2	7.5	0.04472136	0.1	69	7.8	2.49198716	0.3	7.6	0.05477226	0.1	Acti (moderate PA, min/day)	26.7	8.3	8.94427	8.1	1	18.7	7.3	10.4766407	5.4	0.70992957	0.297706	0.17
Samuel-Hodge et al. (2009)	IGvCG	12	low	2	minimal intervention	101	7.8	2.00997512	0.2	7.5	0.04472136	0.1	69	7.8	2.49198716	0.3	7.6	0.05477226	0.1	Acti (light PA, min/day)	26.7	52.3	42.0381	50.6	9.32217786	18.7	53.8	44.8998886	43.7	5.14392846	0.326644	0.19
Samuel-Hodge et al. (2009)	IGvCG	12	low	2	minimal intervention	101	7.8	2.00997512	0.2	7.5	0.04472136	0.1	69	7.8	2.49198716	0.3	7.6	0.05477226	0.1	Accelerometer (total PA, h/day)	26.7	12.1	2.68328	11.9	0.10954451	18.7	12.4	1.49666295	11.7	0.08944272	0.369417	0.22

Data for meta analysis: Outcome objectively measured physical activity

Authors	IG_vs_CG	outcome2	Measurement_method	Number_of_RCT_arms	Control_condition	n_IG	mean_pre_IG	SD_pre_IG	SE_pre_IG	mean_post_IG	SD_post_IG	SE_post_IG	n_CG	MW_pre_CG	SD_pre_CG	SE_pre_CG	mean_post_CG	SD_post_CG	SE_post_CG
Andrews et al. (2011)	IGvCG	MVPA (min/day)	Accelero meter (MVPA, min/day)	3	TAU	240	23	17		31	25		93	26	20		26	23	
Bender et al. (2017)	IGvCG	steps/day	Accelero meter (steps/da y) linear	2	Waitinglis t	22	7483	2415		9524	3626		23	6735	2363		7208	2719	
De Greef et al. (2010a)	IGvCG	steps/day	Pedomete r (steps/da y)	2	TAU	6.7	7099	4208		9601	5002		7	5214	3352		5538	3877	
De Greef et al. (2010a)	IGvCG	MVPA (min/day)	Accelero meter (MVPA, min/day)	2	TAU	6.7	39	35		44	38		7	23	29		25	29	
De Greef et al. (2010a)	IGvCG	total PA (min/day)	Accelero meter (total PA, min/day)	2	TAU	6.7	255	87		253	99		7	223	84		246	109	
De Greef et al. (2010b)	IG1vCG	steps/day	Pedomete r (steps/da y)	3	TAU	7	5924	3725		7630	4423		4	4831	2601		5173	3094	
De Greef et al. (2010b)	IG2vCG	steps/day	Pedomete r (steps/da y)	3	TAU	7.3	5114	2503		5951	3191		4	4831	2601		5173	3094	
Eakin et al. (2013)	IGvCG	total PA (min/week)	Accelero meter (total PA, min/week)	2	TAU	151	125.2	114.7		164	160.4		151	120.2	113.9		122	109.1	
Hoechsm ann et al. (2019)	IGvCG	steps/day	Accelero meter (steps/da y)	2	minimal interventi on	18	5785	793		9783	1334		17	5612	1192		6552	1280	
LookAHE AD group (2013 & 2022)	IGvCG	minutes perr	(MET mir	2	minimal interventi on	492	4.38	6.89		4.51	10.7		497	4.27	7.76		4.12	28.24	
Plotnikoff et al. (2013)	IG1vCG	steps/day	Pedomete r (3-day steps total)	3	minimal interventi on	20.7	21279	3498.73873	769	20821	4208.49587	925	13	21409	2758.24673	765	20097	2996.21311	831
Plotnikoff et al. (2013)	IG2vCG	steps/day	Pedomete r (3-day steps total)	3	minimal interventi on	22.6	21338	3617.7527	761	21493	4202.48802	884	13	21409	2758.24673	765	20097	2996.21311	831
Samuel-Hodge et al. (2009)	IGvCG	MPA (min/day)	Acti (moderat e PA, min/day)	2	minimal interventi on	26.7	8.3	5.16720427	1	8.1	5.16720427	1	18.7	7.3	6.05408953	1.4	5.4	2.5946098	0.6
Samuel-Hodge et al. (2009)	IGvCG	LPA (min/day)	Acti (light PA, min/day)	2	minimal interventi on	26.7	52.3	24.2858601	4.7	50.6	22.2189784	4.3	18.7	53.8	25.946098	6	43.7	9.08113429	2.1
Samuel-Hodge et al. (2009)	IGvCG	total PA (h/day)	Accelero meter (total PA, h/day)	2	minimal interventi on	26.7	12.1	1.55016128	0.3	11.9	1.03344085	0.2	18.7	12.4	0.86486993	0.2	11.7	0.86486993	0.2

Data for meta analysis: Outcome self-reported measured physical activity

Authors	IG_vs_CG	outcome2	Measurement_method	Number_of_RCT_arms	Control_condition	n_IG	mean_pre_IG	SD_pre_IG	SE_pre_IG	mean_post_IG	SD_post_IG	SE_post_IG	n_CG	MW_pre_CG	SD_pre_CG	SE_pre_CG	mean_post_CG	SD_post_CG	SE_post_CG
De Greefet al. (2010b)	IG1vCG	MVPA (min/day)	IPAQ (MVPA, min/day)	3	TAU	7	46	43		75	64		4	44	52		27	39	
De Greefet al. (2010b)	IG1vCG	total PA (min/day)	IPAQ (total PA, min/day)	3	TAU	7	113	74		195	106		4	86	72		65	68	
De Greefet al. (2010b)	IG2vCG	MVPA (min/day)	IPAQ (MVPA, min/day)	3	TAU	7.3	60	60		88	100		4	44	52		27	39	
De Greefet al. (2010b)	IG2vCG	total PA (min/day)	IPAQ (total PA, min/day)	3	TAU	7.3	126	86		158	110		4	86	72		65	68	
Glasgow et al. (2012)	IG1vCG	PA EE/week	CHAMPS (weekly caloric expenditure in PA)	3	TAU	120	4302	2552.38712	233	3307	2760.52169	252	57	3915	2219.65132	294	2882	2264.95033	300
Glasgow et al. (2012)	IG2vCG	PA EE/week	CHAMPS (weekly caloric expenditure in PA)	3	TAU	124	3662	2561.17161	230	3174	2839.55983	255	57	3915	2219.65132	294	2882	2264.95033	300
LookAHEAD study group (2013 & 2022)	IGvCG	MET minutes per 100	Paffenbarger Questionnaire	2	minimal intervention	490.5	5.19	6.53		7.44	9.18		495	5.21	7.15		5.87	7.45	
Plotnikoff et al. (2013)	IG1vCG	MVPA (min/day)	Godin Leisur Time Exercise Questionnaire (MVPA, min/week)	3	minimal intervention	23.7	164.1	83.7341507	17.2	174.5	97.3652916	20	14	156.7	65.1048385	17.4	137.8	68.4723302	18.3
Plotnikoff et al. (2013)	IG2vCG	MVPA (min/day)	Godin Leisur Time Exercise Questionnaire (MVPA, min/week)	3	minimal intervention	24.3	158.9	84.7874519	17.2	153.8	359.85372	73	14	156.7	65.1048385	17.4	137.8	68.4723302	18.3
