# Supplementary material for: Effects of Lifestyle Interventions to Promote Physical Activity on Physical Activity and Glycated Hemoglobin in Patients with Type 2 Diabetes: a Systematic Review and Meta-Analysis
Source: Sports Med. 2025 Mar 13;55(5):1165–81. doi: 10.1007/s40279-025-02184-8 (PMC12106156; doi:10.1007/s40279-025-02184-8)

Effects of lifestyle interventions to promote physical activity on glycated hemoglobin and physical activity in patients with T2D - Systematic review and meta-analysis

Sports Medicine

Vivien Hohberg [1]

Eric Lichtenstein [1]

Jan-Niklas Kreppke [1]

Cedrine Zanitti [1]

Fiona Streckmann [1]

Markus Gerber [1]

Oliver Faude [1]

1) Department of Sports, Exercise and Health, University of Basel, Switzerland

Online Resource 5

Figure 1

Forest Plot random-effect models for the outcome physical activity without the study from Hoechsmann et al.

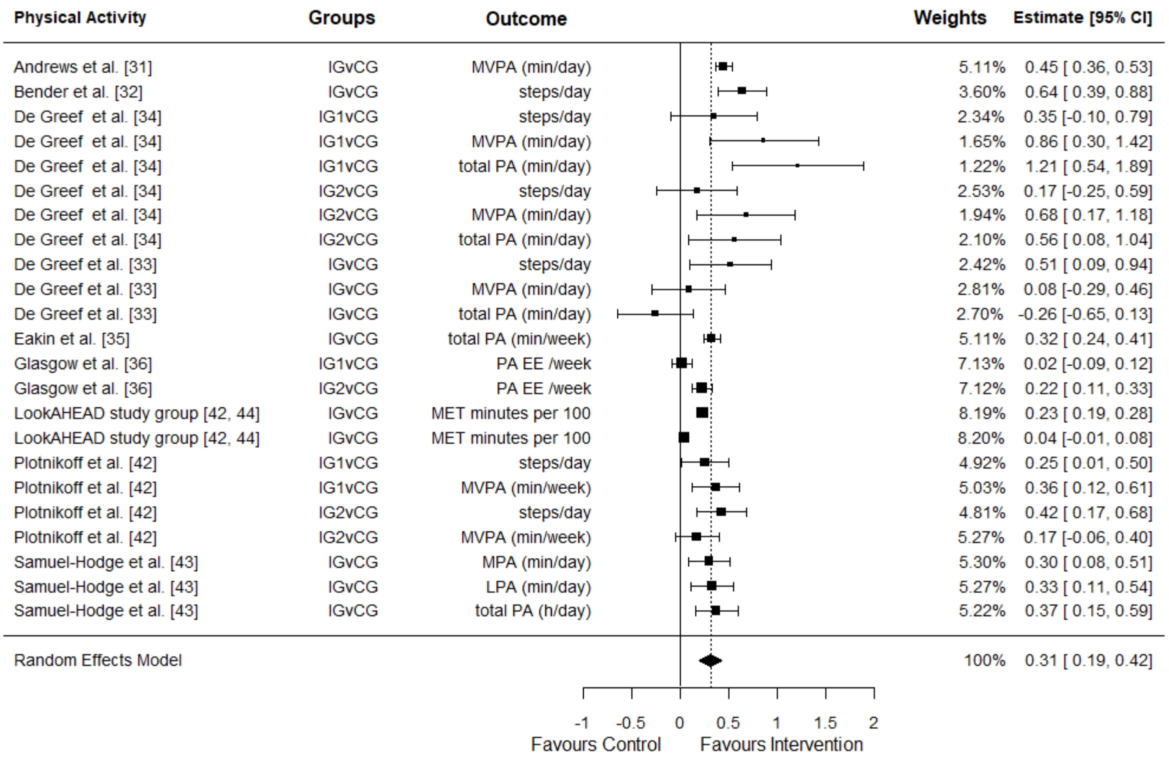

Figure 2

Forest Plot random-effect models for the outcome objectively measured physical activity.

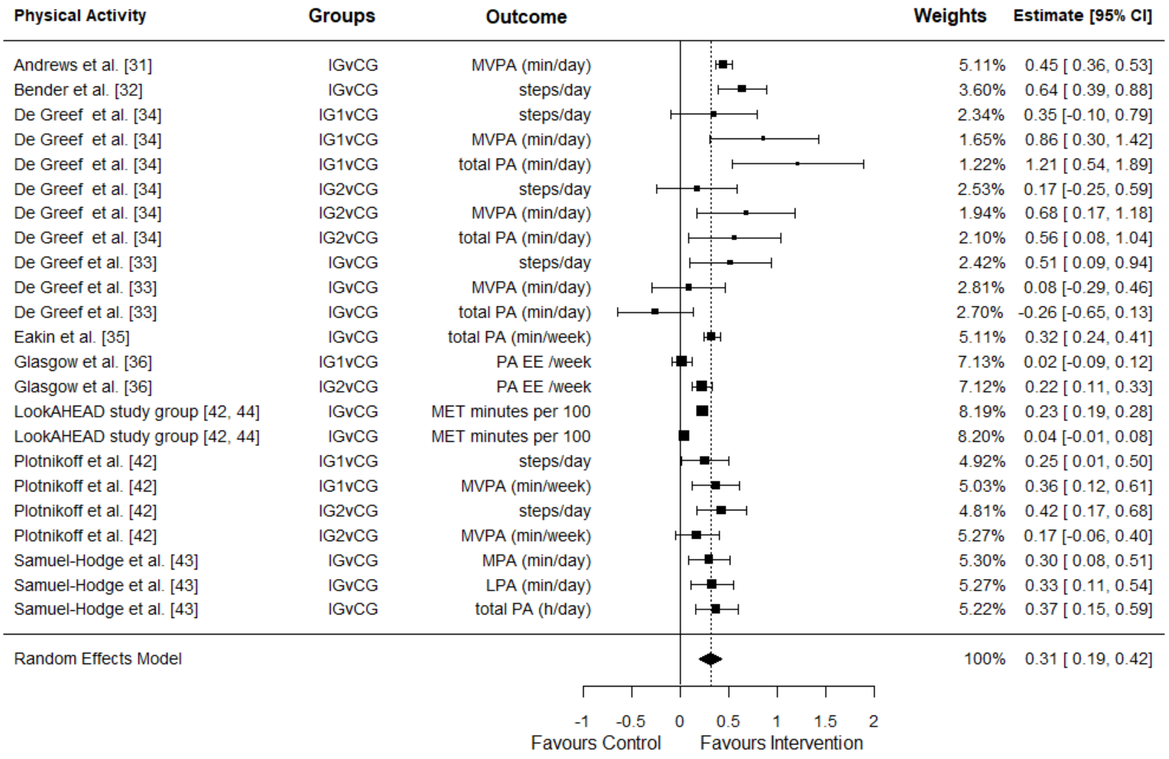

Figure 3

Forest Plot random-effect models for the outcome objectively measured physical activity without the study from Hoechsmann et al.

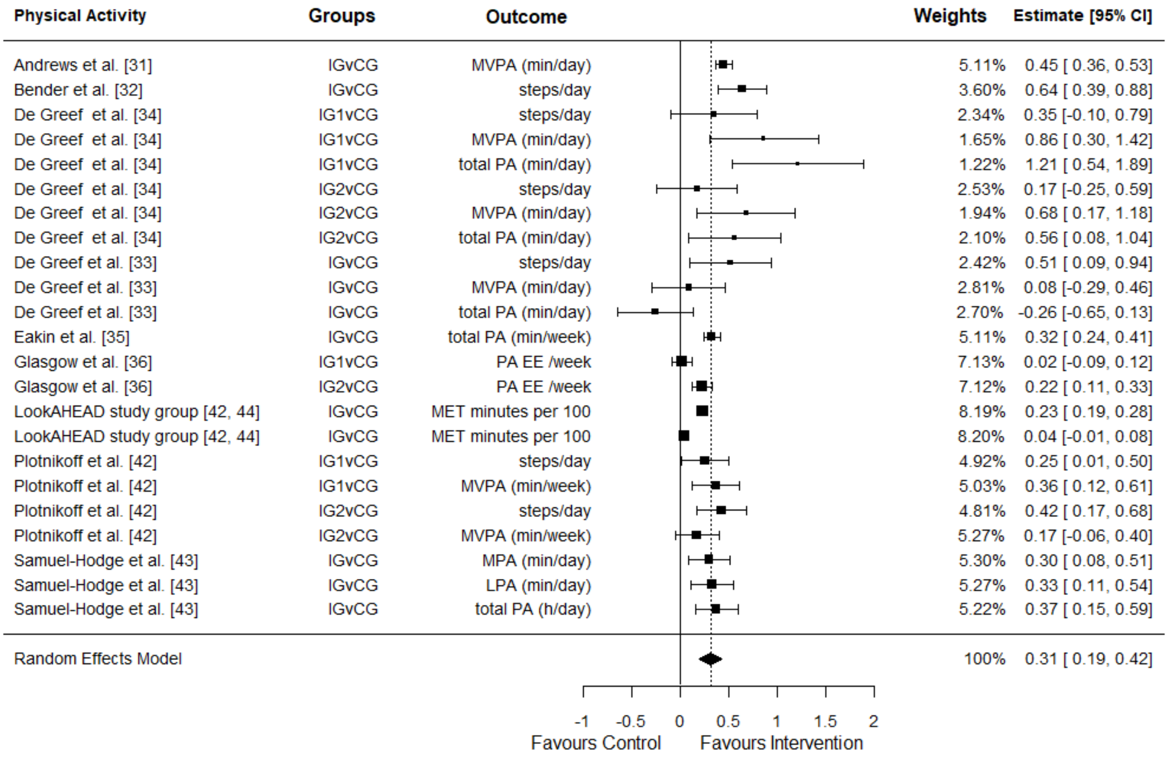

Figure 4

Forest Plot random-effect models for the outcome subjectively measured physical activity

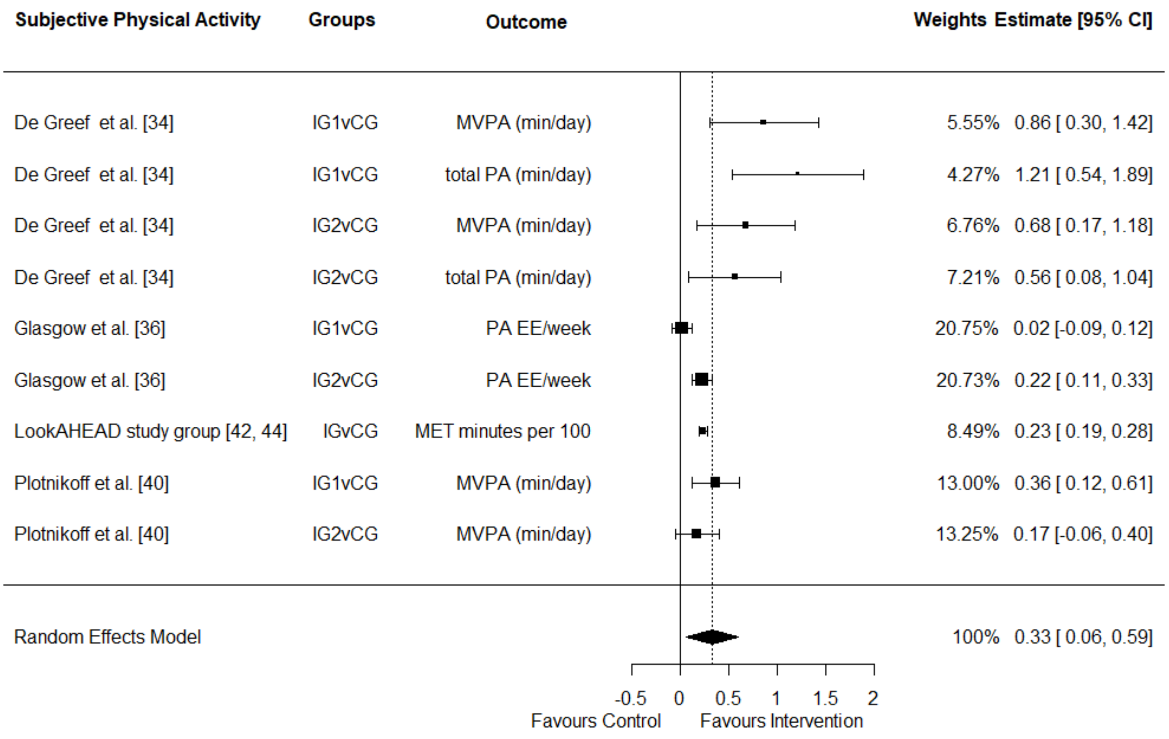

Supplement: Supplementary file 3 — Supplementary file3 (PDF 214 KB) [file 40279_2025_2184_MOESM3_ESM.pdf]
